# Targeting the AMP-activated protein kinase pathway: the active metabolites of botanical drugs represent potential strategies for treating metabolic-associated fatty liver disease

**DOI:** 10.3389/fphar.2025.1681880

**Published:** 2026-01-07

**Authors:** Qi Liang, Li Ai, Lin He, Liang-Chen Wang, Jiao-Long Wang

**Affiliations:** 1 School of Pharmacy, School of Ethnic Medicine, Chengdu University of Traditional Chinese Medicine, Chengdu, China; 2 Chengdu Pidu District Hospital of Traditional Chinese Medicine, Chengdu, China; 3 Sichuan Institute for Drug Control, Chengdu, China

**Keywords:** AMPK, botanical drugs, MAFLD, mechanism, metabolic diseases

## Abstract

AMP-activated protein kinase (AMPK) is capable of regulating cellular energy homeostasis and mitochondrial homeostasis. The activation of AMPK can ameliorate metabolic-associated fatty liver disease (MAFLD). An increasing number of studies have demonstrated that AMPK is a crucial enzyme in the regulation of glucose and lipid metabolism in the body, and numerous botanical drugs act as AMPK activators. This activation can alleviate glucose and lipid metabolism disorders, reduce oxidative stress, and serve as a pivotal therapeutic target for metabolic diseases. MAFLD represents the liver-related manifestation of metabolic syndrome. Currently, there are well - established antidiabetic drugs in clinical practice, yet they also entail side effects. A substantial number of *in vitro* and *in vivo* experimental studies have indicated that natural traditional Chinese medicine targeting AMPK holds promising prospects in the treatment of MAFLD. In this paper, literature was retrieved through electronic databases, including the Web of Science, PubMed, Google Scholar, Springer, and CNKI (Chinese). Therefore, with AMPK as the target, this article analyzes and summarizes the process and mechanism by which botanical drugs activate AMPK to improve glucose and lipid metabolism disorders and mitochondrial homeostasis disorders, aiming to offer a broader perspective for the development of botanical drugs for MAFLD.

## 1 Introduction

Metabolic-associated fatty liver disease (MAFLD), formerly known as non-alcoholic fatty liver disease (NAFLD), is a chronic liver condition marked by fat accumulation, inflammation, and fibrosis, driven by metabolic disorders (Li H. et al., 2025; Zhang Q. et al., 2025). MAFLD is closely linked to the development and progression of liver cirrhosis, hepatocellular carcinoma, obesity, type 2 diabetes, cardiovascular diseases, and various other metabolic disorders. The prevalence of MAFLD is as high as 25% and is increasing year by year (Thapa et al., 2021). MAFLD has emerged as the most prevalent chronic liver disease globally, presenting considerable risks to patient health and placing a significant burden on healthcare systems. Unfortunately, the pathogenesis of MAFLD is multifaceted, and its development is associated with a range of pathological factors, such as disturbances in glucose and lipid metabolism, insulin resistance, mitochondrial dysfunction, oxidative stress-induced damage, and inflammatory processes. At present, hypoglycemic drugs such as metformin, GLP-1 agonists, SGLT-2 inhibitors and recently Resmetirom have significant efficacy and have been well used in clinical practice, but the common side effect of hypoglycemic drugs is hypoglycemia. α-glucosidase inhibitors, biguanides, and insulin sensitizers do not generally cause hypoglycemia when used alone, but they can occur when combined with other drugs. Patients may have a strong feeling of fasting, cold sweat, general weakness, palpitations, trembling hands and feet, eyes, headache, dazed and other phenomena, and coma will occur in severe cases. There is an urgent need to find and develop pharmacological interventions for the treatment of MAFLD (Silva Figueiredo et al., 2018).

AMP-activated protein kinase (AMPK) serves as a pivotal regulator in the control of eukaryotic metabolic pathways. It can be activated under conditions of low cellular energy and functions to maintain energy homeostasis by promoting ATP production while simultaneously inhibiting ATP consumption, a process that is linked to the development of MAFLD (Hsu et al., 2022; Peng et al., 2024). Activation of AMPK can play a role in regulating glucose and lipid metabolism, oxidative stress (OS), inflammation, autophagy and mitochondria. Therefore, AMPK-based MAFLD targeted therapy is feasible (Zhao and Saltiel, 2020). More and more studies have shown that the treatment of MAFLD with natural traditional Chinese medicine not only focuses on reducing blood glucose, but also pays attention to the prevention and treatment of complications. It has high safety and low toxicity, and plays a role in improving the quality of life and prolonging the life span. These drugs can reduce blood lipids, blood glucose, and OS and inflammatory response through multiple pathways (Steinberg and Hardie, 2023). This article reviews new clues for the treatment of MAFLD with botanical drugs targeting AMPK.

## 2 AMPK signaling pathway

### 2.1 The definition and structure of AMPK

AMPK is a serine/threonine protein kinase composed of three subunits that is conserved across eukaryotic organisms. It is composed of the catalytic subunit α (α1, α2), the regulatory subunit β (β1, β2), and the γ subunit (γ1, γ2, γ3). The three different subunits form 12 kinds of αβγ AMPK complexes in a 1:1:1 ratio and have tissue specificity (Khan and Frigo, 2017; Ross et al., 2016).

**FIGURE 1:**
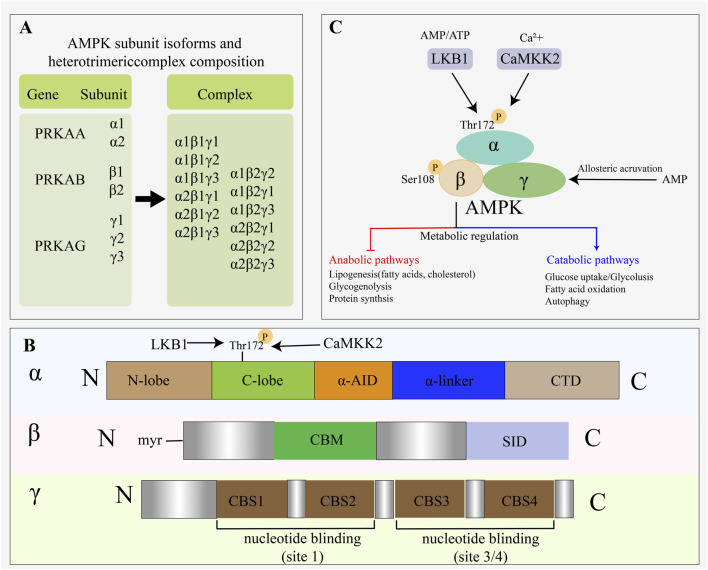
Structure and regulatory activators of AMPK. (**(A)** AMPK subunit isoforms and complex composition; **(B)** domain organization of AMPK; **(C)** LKB1 and CaMKK2 are two direct upstream kinases that activate AMPK).

The α subunit includes the kinase domain that can be phosphorylated by the upstream kinase threonine kinase 172 (Thr172), the auto-inhibitory domain, and the domain for binding to the β subunit, including the carbohydrate-binding molecule and the αγ binding domain, which enables AMPK to bind to glycogen and interact (Juszczak et al., 2020). The γ subunit contains four tandemly repeated cystathionine β-synthase motifs, which activates AMPK after binding to AMP. AMPK has four adenine nucleotide binding sites (Site1 to Site4), and a pair of tandem cystathionine β-synthase forms a “Bateman domain” providing two adenine nucleotide binding sites (Chun and Kim, 2021). After AMP binds to Site3, the KD domain on the α subunit releases the auto-inhibitory domain, converting AMPK to an active conformation (Figures 1A,B).

### 2.2 Factors regulating the activation of AMPK

The activation of AMPK is regulated by varieties mechanisms. AMPK in different subunits is activated by the Thr172 phosphorylation pathway in the α subunit. Liver kinase B1 (LKB1) and calmodulin-dependent protein kinase 2 (CaMKK2) are two well-established upstream kinases capable of activating AMPK. CaMKK2 activates AMPK independent of AMP/ATP ratio (Dengler, 2020). When intracellular Ca^2+^ concentration is increased, CaMKK2 phosphorylates Thr2 to activate AMPK (Zhang et al., 2021). AMP promotes Thr172 phosphorylation to activate AMPK during glucose starvation, which plays a role in sensing energy status and regulating metabolic homeostasis.

As mentioned above, AMPK activity is regulated by nutrient availability such as lipids, amino acids, and carbohydrates, and the expression and phosphorylation of AMPK in liver and adipose tissue are significantly inhibited with HFD. AMPK activity is also regulated by hormones and cytokines, and insulin can inhibit AMPK activity by phosphorylating Ser485 in adipocytes. In addition, tumor necrosis factor α (TNF-α) inhibited AMPK phosphorylation: (1) TNF-α induced PP2C expression to dephosphorylate Thr172 (Kopietz et al., 2020); (2) TNF-α inhibited the activation of AMPK by phosphorylating Ser459 and Ser476 of TBK1 in adipocytes (Zhao et al., 2018) (Figure 1C).

## 3 The role of AMPK in the pathogenesis of MAFLD

### 3.1 Pathogenesis of MAFLD

**FIGURE 2:**
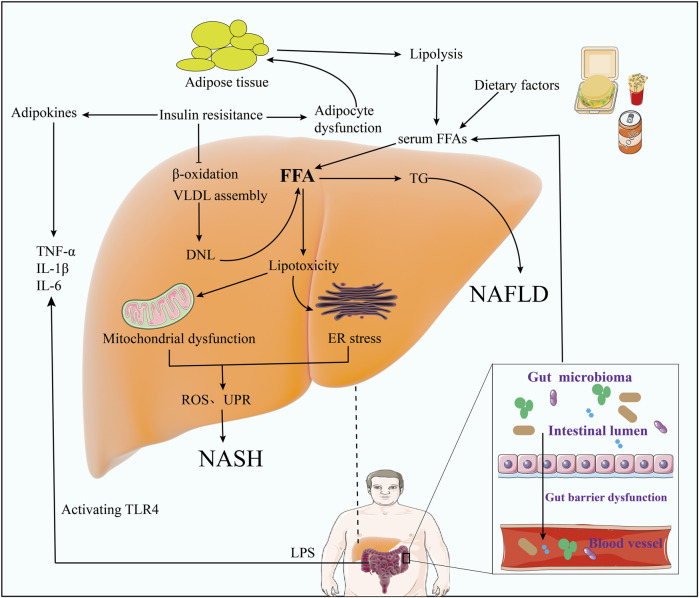
MAFLD’s multiple hit doctrine. (Diet, environment, and obesity lead to elevations in serum fatty acids and cholesterol, inducing insulin resistance, adipose tissue dysfunction, and changes in the gut microbiome).

MAFLD is a complex disease with multiple genetic, epigenetic, and environmental associations, and its pathogenesis is still not fully understood. Currently, the widely accepted theory is the “multiple hit” theory based on the “second hit” theory (Peverill et al., 2014). In the “second-hit” theory, first-hit HFD, obesity, and insulin resistance (IR) lead to hepatic fat accumulation and degeneration, causing inflammation and cell death through a second-hit stimulus. This stimulation will promote OS and eventually lead to steatohepatitis and fibrosis (Rinella et al., 2023), driving NAFLD progression from simple steatosis to non-alcoholic steatohepatitis (NASH). However, in fact, only 5%–20% of patients with NAFLD will develop into NASH, and 10%–20% of patients with NASH will develop into liver fibrosis. Liver fibrosis can transform into cirrhosis, and even cirrhosis can develop into hepatocellular carcinoma (Buzzetti et al., 2016). Thus, the new theoretical “multiple-hit” theory suggests that multiple factors such as IR, lipotoxicity, adipose tissue dysfunction, mitochondrial dysfunction, inflammation, gut microbes, genetic factors, and epigenetic factors work together to drive MAFLD (Figure 2). In this review, we will focus on AMPK and lipid metabolism, IR, inflammatory response, Dysbiosis of intestinal flora to provide a specific review.

### 3.2 AMPK regulates lipid metabolism

The important feature of MAFLD is excessive accumulation of hepatic lipids. When the rates of free lipid uptake, dietary lipid absorption, carbohydrate-mediated *de novo* lipogenesis (DNL), and the conversion and secretion of cholesterol to bile acids exceed those of fatty acid β-oxidation, VLDL-triglyceride (VLDL-TG) secretion, and cholesterol utilization for bile acid synthesis, it results in a gradual accumulation of triglycerides and cholesteryl esters (Donnelly et al., 2005; Li Y. et al., 2011). As a metabolic regulatory center, AMPK responds to the energy level of cells and maintains energy homeostasis. It plays a crucial role in modulating downstream lipid synthesis and lipid metabolism signaling pathways, thereby reducing hepatic lipid accumulation through the inhibition of DNL and cholesterol synthesis, while promoting fatty acid β-oxidation.

**FIGURE 3:**
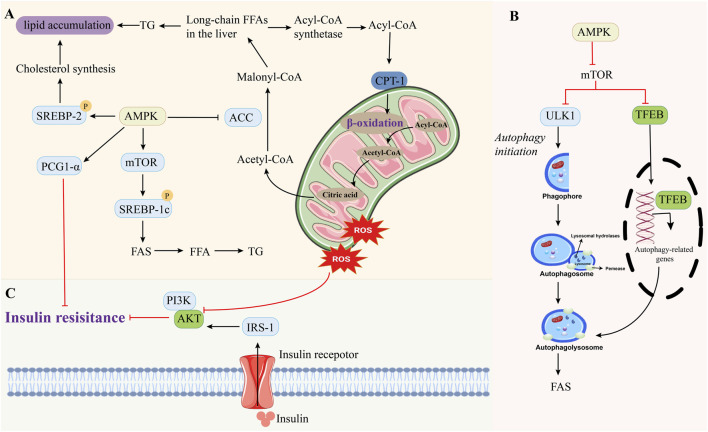
Molecular regulatory mechanisms of AMPK on lipid metabolism, autophagy and insulin resistance. (**(A)** Activation of AMPK directly and indirectly promotes fatty acid entry into mitochondria for β-oxidation; **(B)** AMPK promotes lipolysis through ULK1 or TFEB to promote autophagy; **(C)** AMPK improves insulin resistance by regulating PCG1-α or IRS-1).

Acetyl-CoA carboxylase (ACC), a well-established downstream target of AMPK, facilitates the carboxylation of acetyl-CoA to form malonyl-CoA (Calle et al., 2021). Moreover, ACC1 was phosphorylated at Ser80 and Ser81, and ACC2 was phosphorylated at Ser219 and Ser220, leading to reduced AMPK activity, inhibition of fatty acid synthesis, promotion of β-oxidation, and decreased lipid accumulation. In addition, AMPK can inhibit the downstream targets such as sterol regulatory element-binding protein-1c (SREBP-1c) and rapamycin target (mTOR), reduce lipid synthesis, promote autophagy and β-oxidation, and reduce hepatic steatosis in MAFLD (Figures 3A,B).

During fatty acid oxidation, fatty acids in the cytoplasm undergo an activation step by CoA in the presence of acyl-CoA synthetase to form acyl-CoA. This complex is then transported into the mitochondria via carnitine palmitoyl transferase 1 (CPT1) for subsequent β-oxidation. AMPK reduces the level of malonyl- CoA by inhibiting ACC activity, thereby attenuating the effect of CPT1 (Dobrzyn et al., 2004).

mTOR is a conserved protein kinase that regulates cell metabolism, growth, and autophagy. More and more studies have found that both AMPK and mTOR can regulate autophagy to maintain cell homeostasis (Park et al., 2021). When energy is sufficient, mTOR promotes anabolism and inhibits autophagy, while AMPK promotes cell catabolism and autophagy when energy is short. Moreover, AMPK phosphorylation can directly inhibit mTOR to regulate autophagy (Gosis et al., 2022). Studies have demonstrated that exercise and diet control can increase mTOR phosphorylation, increase Thr172 to promote AMPK activation, increase fat autophagy, and improve lipid accumulation in MAFLD (Gao et al., 2020). In addition, metformin can activate AMPK through LKB1, thereby regulating lipid metabolism in MAFLD.

### 3.3 AMPK regulates IR

IR serves as a critical pathophysiological mechanism in the development of MAFLD. Under normal physiological conditions, insulin exerts precise regulatory control over the hepatic uptake and metabolism of fatty acids (Angelini et al., 2022; Liao et al., 2003). However, when IR is associated with reduced sensitivity to insulin receptors on the surface of hepatocytes, plasma insulin levels increase lipolysis in adipose tissue and induce elevated levels of free cholesterol (FC) in the liver, leading to a large increase in hepatic uptake of fatty acids (Kosmalski et al., 2022). Free fatty acids entering the plasma can inhibit IRS-1 by activating the negative feedback pathway of insulin receptor, and its intermediate products diacylglycerol and ceramide have an inhibitory effect on insulin. Studies have found that diacylglycerol can promote the activation and migration of protein kinase C to the cell membrane, inhibit insulin signaling and aggravate IR. In addition, excessive fatty acids cause excessive cellular energy levels due to β-oxidation, which leads to the inhibition of AMPK activity (Kashyap et al., 2009; Rico et al., 2015). Throughout this process, fatty acids are unable to undergo normal metabolism after entering hepatocytes, leading to the excessive production of reactive oxygen species (ROS). This triggers intracellular inflammatory signaling pathways, further exacerbating lipid accumulation in hepatocytes and ultimately contributing to the development of IR. Studies have shown that β-amino-isobutyric acid can activate AMPK to reduce LPS-induced inflammation and IR in adipose tissue, and has a therapeutic effect on metabolic disorders in MAFLD (Figure 3C).

### 3.4 AMPK regulates inflammatory response

AMPK plays a critical role in modulating inflammatory pathways, and its activation reduces the expression of major inflammatory cytokines such as TNF-α, IL-6, and IL-1β. Activation of AMPK alleviated HFD-induced liver inflammation and fibrosis in mice by reducing the expression of related genes (Garcia et al., 2019).

**FIGURE 4:**
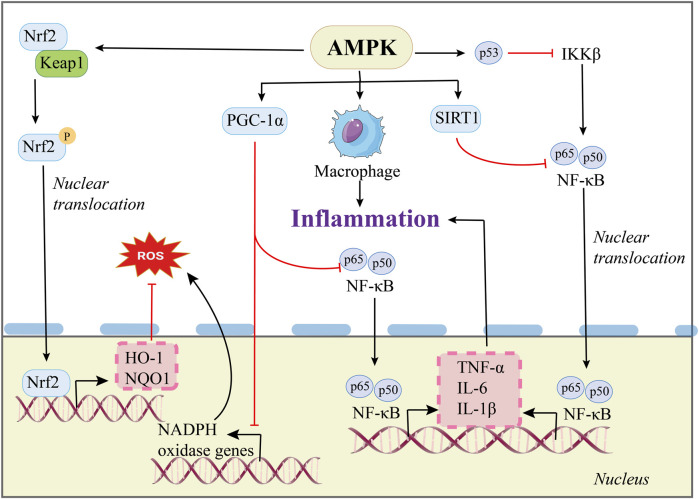
Regulatory mechanisms of AMPK in MAFLD inflammation and OS.

Nuclear factor-κB (NF-κB) is a key regulator of inflammatory responses and controls the expression of genes involved in immune and inflammatory processes. In mice fed a HFD, the expression of the α2 subunit is reduced, while the phosphorylation level of NF-κB is elevated. The use of NF-κB signaling inhibitor can improve metabolic disorders and slow down the progression of steatohepatitis (Zhang et al., 2020). Studies have demonstrated that SIRT1 can deacetylate the p65 subunit of NF-κB, inhibiting its transcriptional activity and reducing hepatic inflammation (de Gregorio et al., 2020) (Figure 4).

Mitochondria are the “energy factories” of cells (Sun Y. et al., 2020). Mitochondrial dysfunction, a process that contributes to increased OS, endoplasmic reticulum (ER) stress, and inflammatory, represents a key pathophysiological mechanism in the progression of MAFLD. Excess lipid beyond the processing capacity of mitochondria or peroxisomes will trigger electron leakage and produce large amounts of ROS, leading to lipid peroxidation (Begriche et al., 2006).

A high-calorie diet and excessive fatty acid intake can exceed the mitochondrial β-oxidation capacity, leading to the accumulation of fatty acids in hepatocytes, which contributes to the development and progression of MAFLD (Zhang X. et al., 2022). In addition, mitochondria are also a key site for the production of ROS in cells. Mitochondrial dysfunction can lead to massive ROS production, triggering OS and ER stress (Goedeke and Shulman, 2021). AMPK serves as a central regulator of cellular energy metabolism. In patients with MAFLD, AMPK is inhibited. Therefore, activation of AMPK negatively regulates target of rapamycin (TORC1), alleviates the inhibitory effect of mTOR on ULK1, and promotes autophagy (Figure 3B), which can stabilize the energy status of cells, thus regulating metabolic homeostasis, balancing lipid metabolism and liver inflammation, and regulating mitochondrial dysfunction. In addition, mitochondrial damage-related molecular patterns (DAMPs) can also activate inflammatory signals, continuously release inflammatory factors, and further trigger oxidative burst, leading to hepatitis and accelerating the progression of MAFLD. In addition, inflammatory factors and ROS induce stromal cells to produce excess collagen, which further induces liver fibrosis (Palabiyik et al., 2016; Salazar-Montes et al., 2008).

### 3.5 Dysbiosis of intestinal flora

Dietary factors and changes in gut microbiota are important causes of MAFLD induction (Bian et al., 2021). Studies show that MAFLD patients have reduced gut microbiota diversity, fewer beneficial bacteria, and increased harmful bacteria. Increased harmful bacteria can induce liver inflammation and OS through the production of endotoxin. In addition, an imbalance in gut microbiota can also influence bile acid metabolism, as these acids undergo microbial-mediated transformation in the intestine following their synthesis in the liver (Cope et al., 2000). Bile acids function as ligands for the farnesoid X receptor (FXR). FXR activation in the liver suppresses DNL, enhances fatty acid β-oxidation, and promotes fibroblast growth factor 21 (FGF21) expression and secretion. This improves insulin sensitivity and reduces gluconeogenesis and IR (Cyphert et al., 2012). Bile acids can also activate G protein-coupled bile acid receptor 5 (TGR5), which induces Kupffer cells to secrete TNF-α through TGR5-camp dependent pathway and inhibits LPS-induced secretion of inflammatory cytokines (Biagioli et al., 2023). Thus, reducing inflammation and protecting MAFLD. In addition to its effects on the liver, activation of TGR5 also ameliorated MAFLD by promoting GLP-1 secretion in intestinal epithelial cells (Chiang and Ferrell, 2020).

## 4 Botanical drugs for MAFLD targeting AMPK

In recent years, the existing drugs for the treatment of MAFLD have exhibited certain limitations, while the incidence of the disease remains high. Therefore, there is an urgent need to explore novel therapeutic approaches. Botanical drugs and metabolites, which are naturally derived, represent a promising and abundant resource for drug development due to their wide availability and relatively low incidence of undesirable effects (Cai et al., 2025; Huang et al., 2021; Qian et al., 2024).

### 4.1 Herbal formulas

According to TCM theory, MAFLD is mainly caused by “qi deficiency”, “phlegm-damp” and “blood stasis” (Dai et al., 2021; Ding et al., 2024), Chinese medicine master and academician Wang Qi developed the empirical Chinese medicine prescription known as Qigui Jiangzhi Formula (QGJZF) (China invention patent, ZL 20210101030103.9), contains *Astragalus membranaceus* Fisch. ex Bunge, *Atractylodes lancea* (Thunb.) DC., *Alisma orientale* (Sam.) Juz., *Crataegus pinnatifida* Bunge, *Cinnamomum cassia* (L.) J.Presl*, Citrus reticulata* Blanco*, Gynostemma Pentaphyllum* (Thunb.) Makino*, Cassia obtusifolia* L. was 30:15:12:20:10:15:20:15., which aims to tonify qi and yang, resolve phlegm-damp, and promote blood circulation. Zhang L. et al. (2025) orally administered QGJZF at doses of 22.8 g/kg and 45.6 g/kg to high - fat diet (HFD) - induced golden hamsters. The experimental results indicated that QGJZF effectively reduced hepatic lipid deposition, and this effect might be mediated via autophagy - related mechanisms. Specifically, QGJZF activates the AMPK/SIRT1 - TFEB pathway, thereby enhancing autophagy and promoting lipid metabolism, which contributes to the improvement of metabolic - associated fatty liver disease (MAFLD). Nevertheless, this research has certain limitations. The composition of traditional Chinese medicine metabolites is highly complex, and only a small number of metabolites have been verified, while the synergistic mechanism of multiple metabolites remains unclear. The detection of autophagic flux is incomplete, and there is a lack of direct evidence regarding lysosomal function. Mechanistic studies primarily focus on hepatocytes, without taking into account the interactions among multiple organs, such as those involving gut microbiota and inflammation. The long - term safety and clinical translation potential have not been assessed. In the future, it is essential to conduct multi - omics integrated analysis, more systematic autophagy monitoring, research on multi - organ interactions, and pre - clinical toxicology and pharmacokinetic investigations.

*Ganoderma lucidum* spore powder, which consists of the mature germ cells of *Ganoderma lucidum* (Leyss. ex Fr.) Karst., is a medicinal and edible fungus that has been utilized in China for centuries to promote health and longevity (Chen et al., 2022; Leng et al., 2023). *Ganoderma lucidum* exhibits protective effects against a variety of liver diseases. Previous studies have found that intragastric administration of *Ganoderma lucidum* Spore Powder can inhibit CCl_4_-induced liver fibrosis and reduce subacute alcoholic liver injury. *Ganoderma lucidum* Spore Powder can also protect mice from obesity induced by high fat intake through lipid metabolism. On this basis, Zhang et al. demonstrated that *Ganoderma lucidum* Spore Powder could effectively reduce lipid accumulation and ROS, *Ganoderma lucidum* Spore Powder was found to alleviate MAFLD by activating AMPK and then activating autophagy and reducing OS in HepG2 cells (Zhang et al., 2024). However, the research has certain limitations. First, although polysaccharides and triterpenoids are mentioned as active metabolites, the effects of specific monomeric metabolites have not been isolated and verified, leading to a vague attribution of the mechanism. Second, only PA was used for induction *in vitro*, failing to simulate the complex lipotoxic environment in MAFLD. Third, autophagy detection only relied on LC3 - II/I and the inhibitor 3 - MA, lacking more rigorous evidence such as lysosomal function and full - process monitoring of autophagic flux. Fourth, long - term safety and clinical translation potential were not evaluated. In the future, studies on metabolite identification, multi - model verification, dynamic monitoring of autophagy, and pre - clinical toxicology need to be carried out.

Wulingsan is composed of *Polyporus umbellatus* (Pers.) Fries, *Poria cocos* (Schw.) Wolf, *Alisma orientale* (Sam.) Juz.*, Atractylodes macrocephala* Koidz.*, Cinnamomum cassia* (L.) J.Presl. Recent studies have demonstrated that various extracts and bioactive monomers derived from Wulingsan exhibit anti-inflammatory and anti-OS properties. Furthermore, as previously mentioned, Ganoderma lucidum has been shown to exert regulatory effects on MAFLD. Therefore, Biao et al. administered three distinct doses of Wulingsan (0.777, 1.554, 3.108 g/kg) to treat rats with MAFLD over a six - week period. The results indicated that Wulingsan activated the AMPK/mTOR/ULK1 pathway, resulting in a notable improvement in liver injury, lipid metabolism dysfunction, oxidative stress (OS), and the inflammatory response. Additionally, it promoted autophagy, thus alleviating MAFLD (Biao et al., 2024). Nevertheless, the metabolites in this study were intricate, and no active monomers were isolated and identified. Moreover, only in - vivo animal experiments were conducted, without validating the mechanism using *in vitro* cell models. In the mechanistic investigation, reliance was placed on static markers such as LC3 - II/I and p62, and the dynamic process of autophagic flux and the direct causal relationship between autophagy and antioxidant activity were not monitored. In general, herbal formulas exhibit considerable potential in the treatment of MAFLD. However, their metabolites are complex, the research model is restricted, and there is a lack of direct causal evidence. In future research, the metabolites should be more comprehensively characterized to offer a more robust assessment for clinical translation. Other herbal formulas are presented in Table 1.

**TABLE 1:** Botanical drugs extracts treat MAFLD by regulating AMPK.

Extracts	Sources	Biological activity	Effective dose	Animals/Cells	Controls	Refs
QGJZF	*Astragalus membranaceus* Fisch. ex Bunge *Atractylodes lancea* (Thunb.) DC. *Alisma orientale* (Sam.) Juz *Crataegus pinnatifida* Bunge *Cinnamomum cassia* (L.) J.Presl *Citrus reticulata* Blanco *Gynostemma Pentaphyllum* (Thunb.) Makino *Cassia obtusifolia* L	Activating AMPK/SIRT1-TFEB Reducing liver lipid deposition and lipid droplets	22.8, 45.6 g/kg, once a day	Male golden hamsters	Positive: atorvastatin calcium (3 mg/kg) Negative: normal diet	Zhang L. et al. (2025)
*Ganoderma lucidum* Spore Powder	Ganoderma lucidum (Leyss. ex Fr.) Karst	Activating AMPK and autophagy Increasing SOD and CAT Reducing lipid accumulatio and ROS	3%, 16 weeks 800 μg/mL	C57BL/6 mice HepG2 cells	Positive: simvastatin (15 mg/kgb.w/day, 16 weeks) Negative: normal diet	Zhang et al. (2024)
Wulingsan	Polyporus umbellatus (Pers.) Fries Poria cocos (Schw.) Wolf *Alisma orientale* (Sam.) Juz *Atractylodes macrocephala* Koidz *Cinnamomum cassia* (L.) J.Presl	Improving liver injury, lipid metabolic dysfunction, OS, and inflammatory Increasing LC3B-Ⅱ, Beclin1, pAMPK and ULK1	0.777,1.554, 3.108 g/kg	SD rats	Positive: PPC (0.144 g/kg) Negative: normal diet	Biao et al. (2024)
Si-Ni-San	*Bupleurum chinense* DC. *Paeonia lactiflora* Pall *Citrus aurantium* L *Glycyrrhiza uralensis* Fisch. ex DC	Reducing lipid accumulation Downregulating fatty acid synthase Activating AMPK/p300/SREBP-1c	2 or 4 g/kg, 4 weeks 200, 400 μM	SD rats HepG2 cells	Positive: metformin (150 mg/kg for 4 weeks) Negative: normal diet	Zhang et al. (2023)
Gypenosides	*Gynostemma pentaphylla* (Thunb.) Makino (Cucurbitaceae)	Reversing the decrease in the ratio of p-AMPK to AMPK Decreasing glucose tolerance	0, 50, 100, 150 and 200 μM 300 mg/kg, 18 weeks	Caco-2 cells Wistar rats	Negative: normal diet	Shen et al. (2022)
Raspberry Extract	*Rubus chingii* Hu	Alleviating HFD-induced liver injury, hepatosteatosis, inflammation, and IR Activating AMPK/PPARα	1 and 2 g/kg/d	C57BL/6 mice	Positive: polyene phosphatidylcholine, 0.178 g/kg/d) Negative: normal diet	Xu et al. (2025)
Qi Zhu Formula	*Astragalus membranaceus* Fisch. ex Bunge *Atractylodes lancea* (Thunb.) DC. *Polygonum cuspidatum* Siebold & Zucc *Polygonum perfoliatum* L *Lycopus lucidus* Turcz. ex Benth. *Citrus reticulata* Blanco	Improving serum profiles Inhibiting lipid synthesis (via AMPK activation, suppressing SREBP1C/FASN/ACC1) Reducing inflammation Attenuating liver injury	0.53, 1.05, 2.10 g/kg	SD rats AML12 cells	Positive: YiShanFu (13.68 mg/mL) Negative: normal diet	Yang et al. (2025)
Xiaozhi formula	*Nelumbo nucifera* Gaertn *Trichosanthes kirilowii* Maxim *Gynostemma pentaphyllum* (Thunb.) Makino *Psidium guajava* L *Salvia miltiorrhiza* Bunge *Polygonum perfoliatum* L	Activating the AMPK and PPAR pathways to attenuate NALFD progression	2.835, 5.67 g/kg/d	C57BL/6J mice	Positive: rosuvastatin (20 mg/kd/d) Negative: normal diet	You et al. (2024)

### 4.2 Metabolites derive from botanical drugs

An increasing number of scientists have directed their attention on the potential preventive and therapeutic effects of natural products in relation to MAFLD (He et al., 2025). Many natural monomer metabolites have been isolated from natural products and their anti-MAFLD effects have been studied. In the previous section, numerous botanical drugs have been documented to exhibit hepatoprotective, lipid-lowering, glucose-lowering, and anti-inflammatory activities, which hold potential in the prevention and treatment of MAFLD. As a key enzyme involved in the regulation of glucose and lipid metabolism, AMPK has emerged as a promising therapeutic target for the development of drugs targeting MAFLD (Steinberg and Carling, 2019). At present, it has been found that metabolites can activate AMPK, including flavonoids, phenols, terpenoids and alkaloids. This review further summarizes the types and pharmacological activities of metabolites that can activate AMPK, thereby providing insights and theoretical references for the development of anti-MAFLD drugs (Table 2).

**TABLE 2:** Metabolites treat MAFLD by regulating AMPK.

Classification	Metabolites	Chemical structure	Biological activity	Effective dose	Animals/Cells	Controls	Refs
Flavonoids	Luteolin	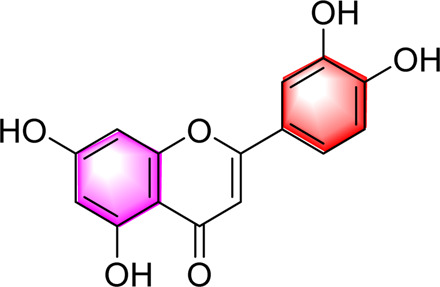	Upregulating AdipoR1, AMPK, and PPAR-γ	50, 100 mg/kg/day, 16 weeks	SD rats	Positive: metformin (100 mg/kg, 16 weeks) Negative: normal diet	Taweesap et al. (2025)
	Isoquercetin	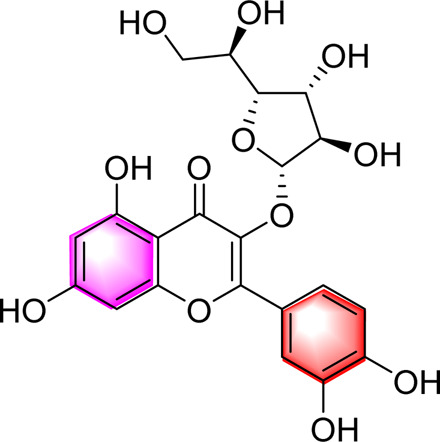	Regulating AMPK/SIRT1/NF-κB Inhibting OS and inflammatory	10, 17.5, 25 mg/kg, 10 weeks	High-fat diet and streptozotocin induced rats	Positive: Rosiglitazone (10 mg/kg, 10 weeks) Negative: normal diet	Qin et al. (2018)
	Cyanidin-3-O-glucoside	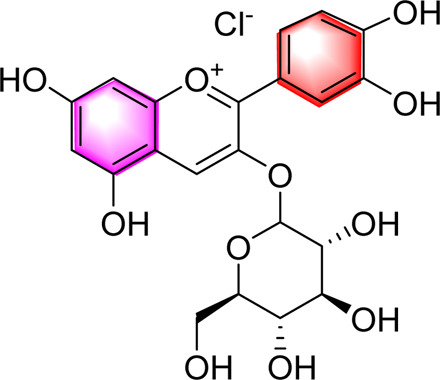	Regulating hepatic lipid homeostasis Activating AMPK	1, 10, 100 μM	HepG2 cell	Positive:/Negative: normal diet	Guo et al. (2012)
	Kaempferol	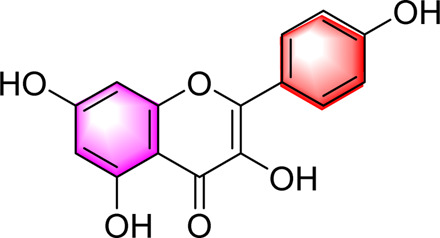	Activating AMPK Regulating lipid metabolism, inflammation and autophagy Reducing apoptosis	10,20,40,80,160 μM	HEPG2 cells	Positive:/Negative: normal diet	Zhou et al. (2022)
	Calycosin-7-O-β-D-glucoside	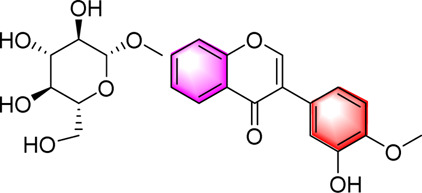	Inhibiting obesity Reducing the disorder of glucose and lipid metabolism, alleviated liver function injury and lipid accumulation Improving the oxidative stress and inflammatory response, AMPK, PPAR-γ, and Nrf2	0.1, 1, 5, 10, 15 µM 5 and 20 mg/kg, 12 weeks	HepG2 cells C57BL/6 mice	Positive: metformin (50 mg/kg, 12 weeks) Negative: normal diet	Zeng et al. (2025)
	Fortunellin	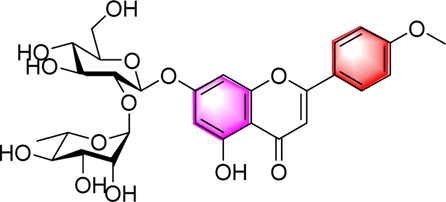	Alleviating liver injury Activating the AMPK	2.5, 5 and 10 mg/kg, once a day for 7 weeks	SD Rats	Positive:/Negative: normal diet	Ping and Qin (2025)
	Baicalein	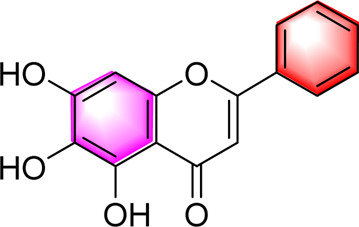	Activating AMPK Inhibiting SREBP1 Reducing hepatic fat accumulation	0, l and 5 μM 50, 200 mg/kg, 3 months	HepG2 cell mice	Positive:/Negative: normal diet	Sun W. et al. (2020)
Phenols	Mangiferin	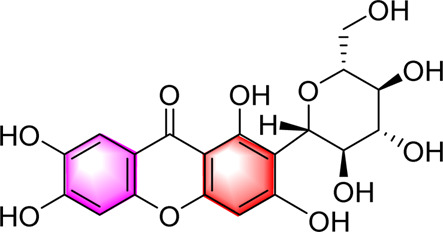	Activating AMPK Regulating sugar, lipid metabolism and anti-inflammatory effect Inhibiting the NLRP3 inflammasome	25, 50, 100 mg/kg/d 400 μM	C57BL/6J mice HepG2 cells	Positive:/Negative: normal diet	Yong et al. (2021)
	Curcumin	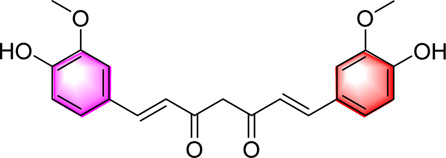	Activating AMPK Improving lipid accumulation in the liver	50 or 150 mg/kg, 6 weeks 0.1, 1, 10, 100 μM	C57BL/6J mice HepG2 cells	Positive: Gemfibrozil (150 mg/kg, 6 weeks) Negative: normal diet	Sun et al. (2021)
	Vanillic acid	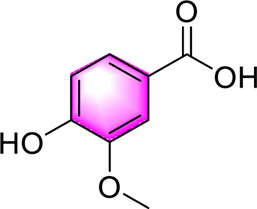	Activating AMPK Inhibiting the production of fat	—	High-fat diet induces mice and db/db mice	Positive:/Negative: normal diet	Shekari et al. (2021)
	Ferulic acid	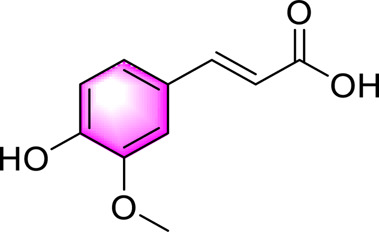	Alleviating OS, liver inflammation and liver fibrosis in the liver Activating PTP1B-AMPK	25, 50, 100 mg/kg, 4 weeks 12.5, 25, 50 μM	C57BL/6J mice LX-2 cells	Positive: CCl4 (1 mL/kg,4 weeks) Negative: normal diet	Wu et al. (2021)
	Chlorogenic acid	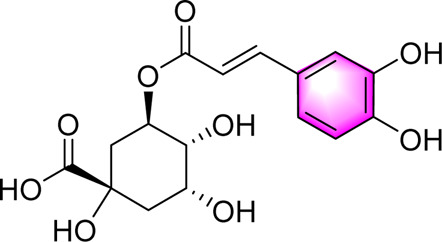	Improving glucose and lipid metabolism Activating AMPK	—	db/db mice; HepG2 cells	Positive:/Negative: normal diet	Ong et al. (2013)
	6-Gingerol	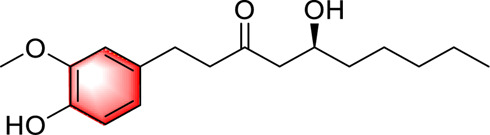	Improving obesity, insulin resistance and hepatic steatosis in mice Reducing lipid accumulation and cholesterol biosynthesis Activating AMPK	100 mg/kg/d 100 μM	C57BL/6 N mice HepG2 cells	Positive:/Negative: normal diet	Xia et al. (2024)
Terpenoids	Nootkatone	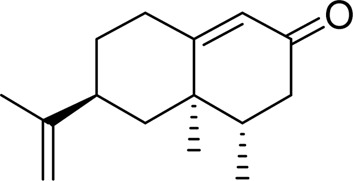	Activating AMP-activated protein kinase Downregulating the phosphorylation of MAPK1/3 (ERK2/1)	25, 100 mg/kg, 4 weeks	C57BL6/J mice	Positive: metformin (200 mg/kg) Negative: normal diet	Li Y. et al. (2025)
			Improving lipid and glucose metabolism disorders, liver injury, and OS in mice Reducing fat accumulation and the deposition of triglycerides Activating AMPK and inhibited MAPK activity Promoting the consumption of glucose and reduces	25, 50 mg/kg, 12 weeks 5,10,20,40 μM	Mice L02 hepatocytes	Positive:/Negative: normal diet	Yong et al. (2022b)
	Actichinone	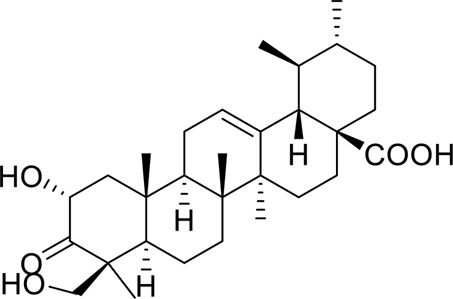	Regulating lipid accumulation and inflammatory response probably via the AMPK/SREBP-1c/PPAR-α and IKK/IκB/NF-κB	10, 20 mg/kg, 18 days	C57BL/6J mice	Positive: OLEA, (20 mg/kg/day, 18 days) Negative: normal diet	Li X. et al. (2025)
	Gypenosides	*Gynostemma pentaphylla* (Thunb.) Makino (Cucurbitaceae	Attenuating hepatic steatosis and intestinal barrier injury in MAFLD rats via the AMPK and TLR4/nuclear factor kappa B (NF-κB) pathways	0, 50, 100, 150 or 200 μM 300 mg/kg, 18 weeks	Caco-2 cell Wistar rats	Positive:/Negative: normal diet	Shen et al. (2022)
​	Ginsenoside CK	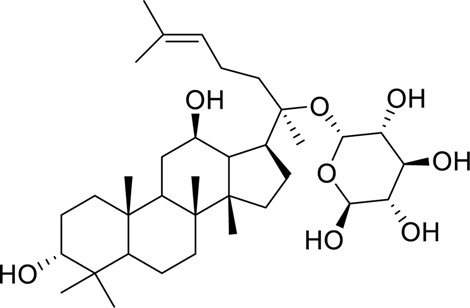	Activation of LKB1/AMPK signaling pathway regulates liver lipid metabolism	1, 2, 5, 10, 15, 20, and 30 μM 30 and 60 mg/kg, 8 weeks	HepG2 cells KM mice	Positive: silibinin (60 mg kg, 8 weeks) Negative: normal diet	Zhang J. et al. (2022)
	HA-20	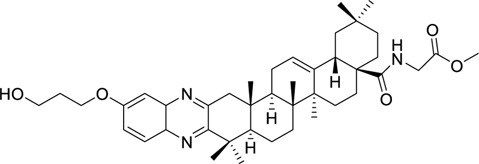	Activating p-AMPK and β-oxidation Reducing hepatocyte steatosis	2.5, 5.0 mg/kg/day 5, 10,20 μM	C57BL/6N mice HepG2, L02, and AML12 cell	Positive: metformin (300.0 mg/kg/day) Negative: normal diet	Wang et al. (2022)
Anthraquinones	Emodin	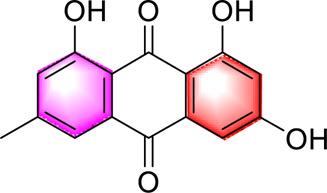	Regulating CaMKK/AMPK/mTOR SREBP1 ameliorates hepatic steatosis	40, 80, 160 mg/kg 20, 40, 80 μM	SD rats HepG2 cells	Positive:/Negative: normal diet	Wang et al. (2017)
	Emodin succinate monoethyl ester	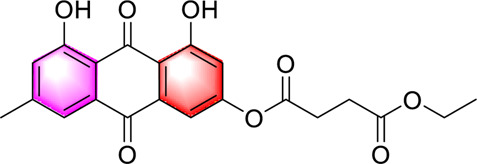	Stimulating AdipoR2 activates CaMKK2 and LKB1 in the liver activate AMPK and PPARα and reduce lipogenesis Reducing lipid deposition and hepatic steatosis	0, 2.5, 10, 40 mg/kg/day, 16 weeks	Syrian hamsters Apoe −/−mice	Positive:/Negative: normal diet	Zhao et al. (2023)
	Danthron	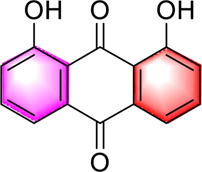	Enhancing hepatic fatty acid oxidation, mitochondrial homeostasis Reducing fat synthesis Activating AMPK/PPARα	10 mg/day/kg, 6 weeks	C57BL/6J mice	Positive:/Negative: normal diet	Ma et al. (2021)
​	Hypericin	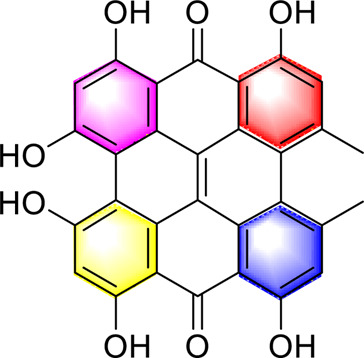	Activation of PKA/AMPK pathway reduces oxidative stress, inhibits lipogenesis and promotes lipid oxidation	20, 200 and 2 μM 0.5, 1 or 2 mg/kg, 3 months	HepG2 cells, L02 cells C57BL/6J mice	Positive:/Negative: normal diet	Liang et al. (2020)
Iridoid glycosides	Geniposide	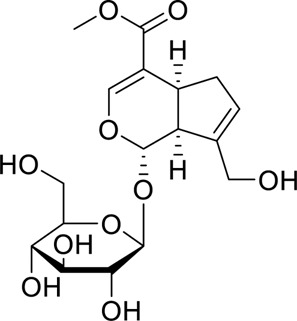	Regulating the Nrf2/AMPK/mTOR Improving the accumulation of lipid in liver cells, OS and inflammatory	65, 130, 260, 390, 520 μM 50, 100 mg/kg, 18 h	HepG2 cells Nrf2^−/−^ C57BL/6 mic	Positive: Fenofibrate (100 mg/kg, 18 h) Negative: normal diet	Shen et al. (2020)
Others	Methyl Cinnamate	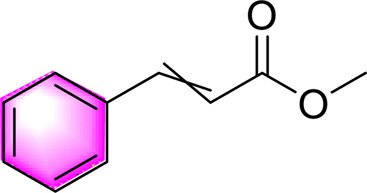	Activating AMPK Improving lipid accumulation in cells	12.5, 25, 50, 100 μM	HepG2 cell	Positive:/Negative: normal diet	Fu et al. (2024)
	Methylsulfonylmethane	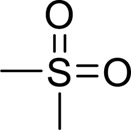	Activating AMPK, ULK1 and insulin sensitivity Inhibiting mTOR, obesity and hepatic steatosis, protein aggregation in mice Reversing damage of autophagic flux	200, 400 mg/kg,9 weeks 50,100,200 nM	C57BL/6 mice HepG2 cells	Positive:/Negative: normal diet	Han et al. (2023)
	Esculetin	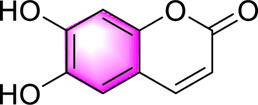	Increasing the fatty acid β-oxidation gene PPAR-α to improve lipid metabolism Activating AMPK Inhibiting JNK, OS, Srebp1c, and lipid accumulation	10,25,50,100,200,500 μM	Wistar rats	Positive:/Negative: normal diet	Xia et al. (2023)
	1,3,6,7-tetrapropylene acyloxy-ketone	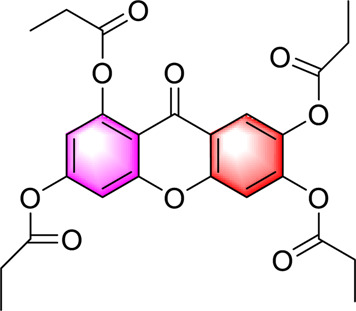	Inhibiting gluconeogenesis Activating AMPK Improving energy homeostasis and insulin resistance	17.5, 35, and 70 μ M	HepG2/HL-7702 cells	Positive: metformin (2 mM) Negative: normal diet	Fan et al. (2023)
	Laurolitsine	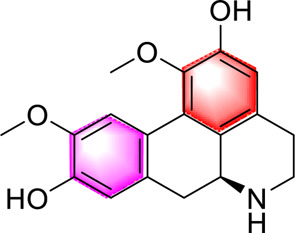	Regulating the LKB1/AMPK Improving the intestinal flora, the metabolism of sugar and lipids	50, 100, 200 mg/kg, 4 weeks 1.25, 2.5, 5 μM	C57BL6/KsJ mice HL-7702 cells	Positive: metformin (200 mg/kg/d, 4 weeks) Negative: normal diet	Yong et al. (2022a)
	Fucoxanthin	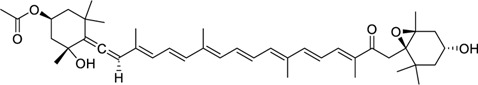	Activating AMPK Regulating the KEAP1/Nrf2/ARE signaling pathway to exert antioxidative effects and stimulating the PGC1α/NRF1 axis to enhance mitochondrial biogenesis	0.25 μM 0.2%, 24 weeks	HepG2 cells ob/ob mice	Positive:/Negative: normal diet	Yang et al. (2024)

#### 4.2.1 Flavonoids

Luteolin is a naturally occurring flavonoid metabolite present in various botanical drugs, fruits, and vegetables, known for its antioxidant, anti-inflammatory, and cardiovascular protective properties (Ahmadi et al., 2020; Yasmin et al., 2021). Luteolin was found to reduce OS in rats with metabolic syndrome (Dai et al., 2025), and in addition, luteolin has been demonstrated to alleviate insulin resistance, a key factor in the management of MAFLD (Dai et al., 2025). Therefore, based on these findings, 4 weeks of oral administration of luteolin at doses of 50 and 100 mg/kg/day was shown to improve metabolic parameters induced by HFD. Luteolin significantly reduced blood pressure, fasting blood glucose levels, serum insulin concentrations, free fatty acid levels, cholesterol levels, and hepatic steatosis. In addition, luteolin upregulated the expression of AdipoR1, AMPK, and PPAR-γ, thereby improving MAFLD (Taweesap et al., 2025). However, this study was exclusively based on animal models, and no control group was set up with a normal diet and luteolin. As a result, it is difficult to rule out the influence of its own metabolic effects, and the conclusions should be extrapolated to humans with prudence. In the future, gene knockout or antagonism experiments can be utilized to verify the necessity of the pathway, and pre-clinical pharmacokinetic/toxicological studies can be carried out.

Isoquercetin, a glucoside derivative of quercetin, is also a flavonoid found in *Cichorium glandulosum Bois.* et Huet (Hoek-van den Hil et al., 2013; Li R. et al., 2011). It has strong antioxidant activity, and several studies have shown that isoquercetin has therapeutic effects on insulin resistance, fat oxidation and hepatic steatosis (Ding et al., 2014; Guo et al., 2013). Qin et al. validated the use of different doses of isoquercetin (10, 17.5, 25 mg/kg) in the treatment of MAFLD induced by HFD *in vivo*. The results demonstrated that HFD-induced MAFLD was significantly ameliorated by isoquercetin, as evidenced by reduced lipid accumulation, inflammation, and OS. Furthermore, isoquercetin activated AMPK and exhibited a crucial role in lipid metabolism regulation. In conclusion, isoquercetin alleviates MAFLD through activating AMPK and the inhibiting TGF-β, leading to improvements in hepatic lipid accumulation and OS (Qin et al., 2018). However, despite proving effective, there are certain limitations. In this study, MAFLD, a metabolic disorder, was investigated with a relatively short treatment duration of 4 weeks. Moreover, molecular docking merely predicts targets without experimental validation. It is recommended that in future research, it is essential to validate whether TGF-βR1/2 is a direct target and explore the interaction between the AMPK and TGF-β pathways.

Cyanidin-3-O-β-glucoside, the main active metabolite of *Morus alba* L., is a typical anthocyanin pigment. Guo et al. studied the effect of Cyanidin-3-O-β-glucoside in HepG2 hepatocytes *in vitro* and found that Cyanidin-3-O-β-glucoside (1, 10, 100 μM) increased AMPK activity in a calomodulin kinase-dependent manner. It can regulate liver lipid homeostasis through AMPK signaling pathway to play an anti-MAFLD effect (Guo et al., 2012). However, this study is only based on liver cancer cell lines, has not been verified in animal or human models, and the experimental time is short, and there is a lack of long-term effect data. Moreover, the bioavailability and synergistic effect of Cyanidin-3-O-β-glucoside in complex food substrates were not investigated. In the future, animal and clinical studies are needed to verify its anti-MAFLD effect *in vivo*.

#### 4.2.2 Phenols

The representative phenolic monomer metabolites in botanical drugs medicines with AMPK activation are mainly mangiferin, curcumin, vanillic acid, ferulic acid, chlorogenic acid, and 6-Gingerol et al.

Mangiferin is a naturally occurring phenolic metabolite predominantly found in mango leaves. Research has indicated that mangiferin exhibits antioxidant properties (Sharma et al., 2024), anti-inflammatory (Saha et al., 2016), anti-tumor (Gold-Smith et al., 2016) and anti-diabetic effects (Yon et al., 2005). In a double-blind, randomized, controlled clinical trial, mangiferin was found to significantly reduce serum triglyceride and free fatty acid levels, thereby improving lipid profiles in overweight individuals with hyperlipidemia. Mangiferin may serve as a promising therapeutic agent for the management of MAFLD. Zhang et al. conducted a comprehensive investigation into the effects and underlying mechanisms of mangiferin in relation to MAFLD, utilizing both *in vivo* and *in vitro* experimental models. The results indicated that mangiferin effectively ameliorated diet-induced hepatic injury, IR, and glucose intolerance in mice. Notably, the study revealed that mangiferin activated AMPK while simultaneously inhibiting NLRP3 inflammasome activation and pyroptosis, findings that were corroborated in subsequent *in vitro* cell experiments. These effects suggest that mangiferin exerts a regulatory effect on glucose and lipid metabolism (Yong et al., 2021). The study duration was brief (12 weeks). The long - term efficacy and potential side effects were not assessed, and the cell experiment solely employed the HepG2 cell line, which did not fully mimic the in - vivo liver cell microenvironment. In the future, it is advisable to conduct long - term and multi - species model experiments, comprehensively explore the interaction network by integrating transcriptomics and proteomics, and advance dose optimization and clinical trials to elucidate its therapeutic potential and safety.

Curcumin is one of the well-studied natural monomer metabolites in *Curcuma longa* L. It is a phenolic metabolite with hepatoprotective, hypoglycemic and antioxidant activities, and has attracted much attention due to its significant potential in the treatment of various diseases. Zhai et al. found that curcumin inhibits liver fibrosis by activating the AMPK pathway to promote PGC-1α expression, increase PPARγ activity and SOD-2 transcription and activity. Curcumin can also inhibit the generation of intracellular ROS, alleviate mitochondrial membrane potential damage, protect mitochondrial function, and thereby reduce hepatocyte apoptosis (Zhai et al., 2015; Sun et al., 2021). However, the study exhibits certain limitations. It failed to explore the specific mechanism and potential role of SLC25A1 (mitochondrial citrate carrier) in MAFLD cases that did not demonstrate significant changes. Additionally, it did not conduct long - term toxicity and pharmacokinetic studies. The clinical dosage and safety of this drug remain to be verified. In the future, it is essential to establish animal models that more accurately mimic the progression of human NASH. In combination with multi - omics technologies, a comprehensive analysis of the citric acid metabolic network should be carried out, and pre-clinical safety and optimal dose optimization studies should be promoted.

Vanillic acid (Shekari et al., 2021) is one of primary bioactive metabolites found in *Coix lacryma-jobi* L. var. *mayuen*, which can activate AMPK to play lipid-lowering, anti-inflammatory and anti-OS. Ferulic acid (Shekari et al., 2021) is widely found in botanical drugs such as *Coix lacryma-jobi* L. var. *mayuen* and *Areca catechu* L., and exhibits a range of biological activities, including antioxidant, anti-inflammatory, and immune-enhancing properties. Wu et al. demonstrated that ferulic acid alleviates OS and hepatic inflammation via the PTP1B-AMPK signaling pathway, thereby improving symptoms associated with MAFLD. Chlorogenic acid (Ong et al., 2013) is one of the representative phenolic metabolites extracted and isolated from the natural drug *Lonicera japonica* Thunb. Previous studies have demonstrated that chlorogenic acid exerts beneficial effects on MAFLD through the activation of AMPK, thereby modulating glucose and lipid metabolism.

*Zingiber officinale* Rosc. contains hundreds of bioactive metabolites (de Lima et al., 2018), among which 6-gingerol is a major bioactive metabolite that has demonstrated significant effects in anti-inflammatory activity, tumor suppression, and the improvement of lipid metabolism (Zhong et al., 2019). An important study found that 6-gingerol promoted adipocyte Browning in adipocytes by activating AMPK, providing a new perspective for obesity treatment. In another study (Ahn et al., 2021; Xia et al., 2022), ban-xia-xie-xin-tang was found to contain ginger, and 6-gingerol was identified as one of its major bioactive metabolites, demonstrating a significant effect in ameliorating HFD-induced hepatic steatosis. Therefore, to gain further insight into the mechanism of action of 6-gingerol. In 2024, Xia et al. showed that oral administration of 6-gingerol at 100 mg/kg/day significantly protected against obesity, insulin resistance, and hepatic steatosis in mice. *In vitro*, 100 μM 6-gingerol reduced lipid accumulation in HepG2 cells. Combined with omics analysis and validation, it was found that 6-gingerol alleviated hepatic steatosis by activating AMPK-SREBPs to reduce hepatic triglyceride and cholesterol biosynthesis. These results suggest that 6-gingerol may serve as a promising therapeutic candidate for the management of MAFLD (Xia et al., 2024). Although this study was validated through *in vivo* and *in vitro* experiments, it was discovered that the short intervention period (12 weeks of high-fat diet (HFD) + 4 weeks of drug administration) did not fully mimic the chronic process of the progression of human metabolic associated fatty liver disease (MAFLD) to non-alcoholic steatohepatitis (NASH)/fibrosis. Moreover, there was a deficiency of long-term toxicity and pharmacokinetics data, and the potential for clinical translation requires evaluation. In the future, liver-specific gene editing models, multi-omics integrated analysis, and longer-term interventions are necessary to comprehensively elucidate its action network and safety.

#### 4.2.3 Terpenoids

Nootkatone is a sesquiterpene ketone that occurs in *Alpinia oxyphylla* Miq. (Wang et al., 2018), grapefruit, and citrus. It possesses pharmacological properties such as spleen-warming and antidiarrheal effects, reduction of saliva secretion, kidney-warming functions, diuresis modulation, and sperm-stabilizing activity. Previous studies have demonstrated that nootkatone effectively suppresses weight gain (Li et al., 2021), and in 2022, Yong et al. (2022b) explored the potential of nootkatone to treat MAFLD. The results showed that nootkatone improved lipid and glucose metabolism disorders and reduced fat accumulation in liver tissue in MAFLD mice. Nootkatone activated AMPK and inhibited MAPK to reduce p-JNK, thereby improving MAFLD *in vivo* and *in vitro*. Immediately following, in 2025, Li Y. et al. (2025) also demonstrated that nootkatone attenuated the progression of MAFLD in a diabetic model by activating AMPK and inhibiting MAPK using db/db mice. However, no pharmacokinetic study was performed in the above experiments. In the future, gender differences and supplementary PK studies are needed to promote further molecular mechanism verification to promote clinical application. In conclusion, nootkatone is a promising natural monomer for the treatment of MAFLD.

As early as 2016 (Lin et al., 2016), it was reported that oleanolic acid had a protective effect on liver. Following this idea, Wang et al. found that oleanolic acid derivative HA-20 could inhibit lipogenesis and reduce hepatic steatosis in 2021 (Wang et al., 2021). In 2022, HA-20 (2.5, 5.0 mg/kg/day) was found to prevent hepatocyte steatosis in MAFLD mouse, and three cell lines HepG2, L02 and AML12 were used to verify that HA-20 could activate AMPK to inhibit lipogenesis and enhance β-oxidation. However, the molecular mechanism by which HA-20 enhanced Ca^2+^ release was not elucidated, such as through calcium channels or endoplasmic reticulum release. Only the early MAFLD model (8 weeks) was used in the animal experiment, and its effect on advanced lesions (e.g., fibrosis, inflammation) was not evaluated. In the future, it is necessary to further reveal its calcium regulatory mechanism, verify its efficacy and safety in longer cycles and more advanced disease models, improve its pharmacokinetics and explore its potential side effects in other organs. In general, HA-20 increases Ca to prevent MAFLD provides new insights (Wang et al., 2022).

#### 4.2.4 Anthraquinones

Emodin succinate monoethyl ester is a new type of anthraquinone metabolite. Zhao et al. used two experimental animals, hamsters and mice, to verify the effect of Emodin succinate monoethyl ester on MAFLD, and the research results showed that, Emodin succinate monoethyl ester activates AdipoR2, AMPK and PPARα and reduces lipid deposition and hepatic steatosis. In this study, emodin succinate monoethyl ester was also compared with statins and emodin. Emodin succinate monoethyl ester demonstrated superior efficacy and safety in alleviating hepatic steatosis and protecting hepatocytes, suggesting its potential as a promising novel therapeutic agent for the clinical management of MAFLD (Zhao et al., 2023). However, our review of the literature found that this study only used male animals and did not investigate gender differences, and the long-term safety assessment was insufficient. In the future, it is necessary to carry out long-term toxicological studies and pharmacokinetic analysis involving both sexes, and to verify the efficacy in models closer to human pathology (such as MAFLD with fibrosis).

#### 4.2.5 Iridoid glycosides

Geniposide, the primary bioactive metabolite of *Gardenia jasminoides* Ellis, has been demonstrated to exhibit hepatoprotective, hypoglycemic. Accumulating evidence suggests its therapeutic potential in the treatment of various diseases, making it a focal point in recent research on chronic metabolic disorders. Shen et al. (2020) demonstrated that geniposide enhances antioxidant and anti-inflammatory capacities and effectively alleviates hepatic lipid accumulation by upregulating the expression of Nrf2/HO-1 and AMPK, thereby inhibiting mTOR and its downstream signaling proteins. Geniposide can also reduce myocardial ischemia and improve diabetic nephropathy by activating AMPK to mediate ULK1, NLRP3 and NF-κB (Dusabimana et al., 2021; Li et al., 2020). However, there are significant limitations in this study. The pathophysiology of tyloxapol-induced acute fatty liver is quite different from that of human chronic MAFLD. The safety and pharmacokinetic profile of long-term administration were not assessed. In the future, it is necessary to verify the efficacy of Geniposide in chronic models closer to human disease characteristics (such as high-fat diet induced), further analyze the molecular targets of geniposide, and carry out systematic pharmacological and safety studies.

#### 4.2.6 Others

In addition to the large groups of metabolites mentioned above, there are Methyl Cinnamate, Methylsulfonylmethane, Esculetin, 1,3,6,7-tetrapropylene acyloxy-ketone and so on. Methyl Cinnamate, a bioactive volatile metabolite derived from *Zanthoxylum armatum* DC (Bhatia et al., 2007), is widely present in food and household products and has been approved by the FDA for use as a flavor agent. *Zanthoxylum armatum* DC was previously found to attenuate HFD-induced fatty liver in mice, and Methyl Cinnamate was found to upregulate AMPK phosphorylation in 3T3-L1 cells *in vitro* (Lilin et al., 2023). Subsequently, Fu et al. investigated the effect of Methyl Cinnamate in HepG2 cells and found that Methyl Cinnamate (12.5, 25, 50, 100 μM) could reduce triglyceride, p-AMPK/AMPK and CPT1a. Improved lipid accumulation in MAFLD (Fu et al., 2024). The study still shows some limitations, including the lack of animal or clinical data to confirm efficacy *in vivo*; However, the mechanism by which AMPK is activated by Methyl Cinnamate remains unclear. Further *in vivo* studies are needed to elucidate the structural basis of the interaction between Methyl Cinnamate and AMPK, and to evaluate its long-term safety and therapeutic potential.

Esculetin, a coumarin metabolite derived from *Fraxinus rhynchophylla* Hance, has anti-tumor, anti-inflammatory, and anti-OS properties. Studies have found that esculetin has an effect on metabolic dysfunction, which can alleviate IR and vascular dysfunction in diabetic rats (Garg et al., 2022; Liang et al., 2017). In previous studies, Esculetin also ameliorated liver inflammation and improve lipid metabolism in animal models induced by metabolic dysfunction factors (Kadakol et al., 2017; Zhang L. et al., 2022). Study investigated the potential of Esculetin (at concentrations of 10, 25, 50, 100, 200, and 500 μM) in the context of MAFLD and demonstrated that esculetin significantly attenuated OS in primary rat hepatocytes. Furthermore, esculetin exhibited promising effects on lipid metabolism in primary hepatocytes exposed to non-lipotoxic oleic acid/palmitic acid (OA/PA) by downregulating the lipogenesis-related gene SREBP1c and upregulating the fatty acid β-oxidation-related PPAR-α. These effects were achieved through activating AMPK and inhibition of JNK, thereby fulfilling its potential as a therapeutic agent for MAFLD (Xia et al., 2023). However, this study is only based on primary rat hepatocytes, and the specific mechanism and metabolic pathway of Esculetin entry into cells have not been elucidated *in vivo*. There is a lack of in-depth analysis of lipid droplet morphology and function under different fatty acid combinations. Long-term safety and potential side effects were not assessed. Future studies in animal models are needed to clarify the bioavailability, tissue distribution, and metabolic fate of Esculetin.

### 4.3 Botanical drugs for MAFLD targeting AMPK

Overall, in addition to maintaining intracellular energy balance, AMPK regulates energy metabolism throughout the body and can be activated in response to decreased cellular energy levels, triggering mitochondria by activating ULK1 and inhibiting mTORC1. AMPK can also protect against MAFLD by regulating glucose and lipid metabolism, promoting the Browning of white adipose tissue, anti-inflammation, anti-oxidative stress, IR, and Dysbiosis of intestinal flora. Although the AMPK signaling pathway has many effects, the current role of botanical drugs in the treatment of MAFLD is mainly focused on inhibiting liver *de novo* lipogenesis, promoting liver fatty acid oxidation and enhancing adipose tissue mitochondrial function. In addition, AMPK also has the effect of improving inflammation (apoptosis, liver fibrosis and autophagy). The pathogenesis of MAFLD is much more than liver steatosis and mitochondrial homeostasis disorders. Whether traditional Chinese medicine monomers can participate in the intervention of “multiple hits” of MAFLD from more aspects through AMPK signaling pathway still needs more extensive research. In addition, many studies have found common problems, such as the lack of long-term experiments and pharmacokinetic observation of drug potency and toxicity. Future research should focus on the above problems in addition to *in vitro* and *in vivo* studies to evaluate the potential of drugs for clinical translation.

## 5 Conclusion and perspectives

MAFLD is a chronic liver disease linked to metabolic disorders. It results from a combination of genetic, environmental, and metabolic factors. The prevalence and economic burden of MAFLD are progressively increasing. More and more studies have confirmed that AMPK is a key target of metabolic diseases. Activating AMPK can regulate insulin resistance, mitochondrial dysfunction, etc., thereby reducing lipid accumulation caused by MAFLD and restoring metabolic disorders *in vivo*, which holds significant therapeutic potential for the treatment of MAFLD.

Currently, several drugs available on the market have demonstrated efficacy in the treatment of MAFLD based on clinical trial data, including AMPK activators, some hypoglycemic drugs and antioxidants (Wu et al., 2025). PXL770 is a novel, direct activator of AMPK that effectively inhibits *de novo* adipogenesis and demonstrates efficacy in preclinical models; it is currently undergoing Phase II clinical trials (Gluais-Dagorn et al., 2022). Metformin is a commonly prescribed biguanide for the management of T2DM. Research indicates that metformin lowers blood glucose levels by improving glycogen metabolism and enhancing glucose uptake in muscle cells through an AMPK-dependent mechanism. However, its safety and efficacy in patients with MAFLD remain a subject of debate (Ma et al., 2022). However, long-term use of these synthetic drugs is often associated with notable adverse effects, such as gastrointestinal disturbances. Notably, botanical drugs medicines and their derived metabolites have garnered considerable attention due to their extensive research background and favorable safety profile, thereby demonstrating significant potential for future clinical application in the management of MAFLD. This article provides a systematic review of the potential of botanical drugs and their monomeric metabolites in the treatment of MAFLD through the regulation of AMPK. However, the development of botanical drugs to treat MAFLD also faces many challenges.

Firstly, as a drug target, AMPK exhibits a complex mechanism of action owing to its extensive systemic distribution. Further research is required to fully evaluate its role in MAFLD and to determine the optimal conditions for its activation. Secondly, most of the current studies on AMPK-targeting botanical drugs against MAFLD are mainly animal and cell experiments. Therefore, more systematic and rigorous randomized controlled human trials are needed to provide a basis for the development of new drugs for botanical drugs and their monomer metabolites. Thirdly, due to the poor properties of some natural medicines, appropriate drug dosage forms should be selected to improve safety and clinical efficacy.

In summary, this review summarizes the anti-MAFLD botanical drugs by targeting AMPK signaling pathway. It also examines the current limitations and future research directions of using botanical drugs as clinical interventions for MAFLD. The aim is to encourage further research and development of botanical drugs therapies, thereby enhancing the understanding and application of these botanical agents.
